# Supplementary material for: Neutralization of SARS-CoV-2 by IgM-14 via engagement of two distinct spike epitopes
Source: PLoS Pathog. 2026 Mar 25;22(3):e1014071. doi: 10.1371/journal.ppat.1014071 (PMC13043055; doi:10.1371/journal.ppat.1014071)
Supplement: S1 Fig — Red circles and curves represent response to IgM-14 treatment; open black circles with solid dark curves indicate response to IgG-14 treatment. USA-WA1/2020 has been tested for four different batches, each with three replicates. Means from three independent experiments are shown. Error bars indicate standard deviations. (DOCX) [file ppat.1014071.s001.docx]

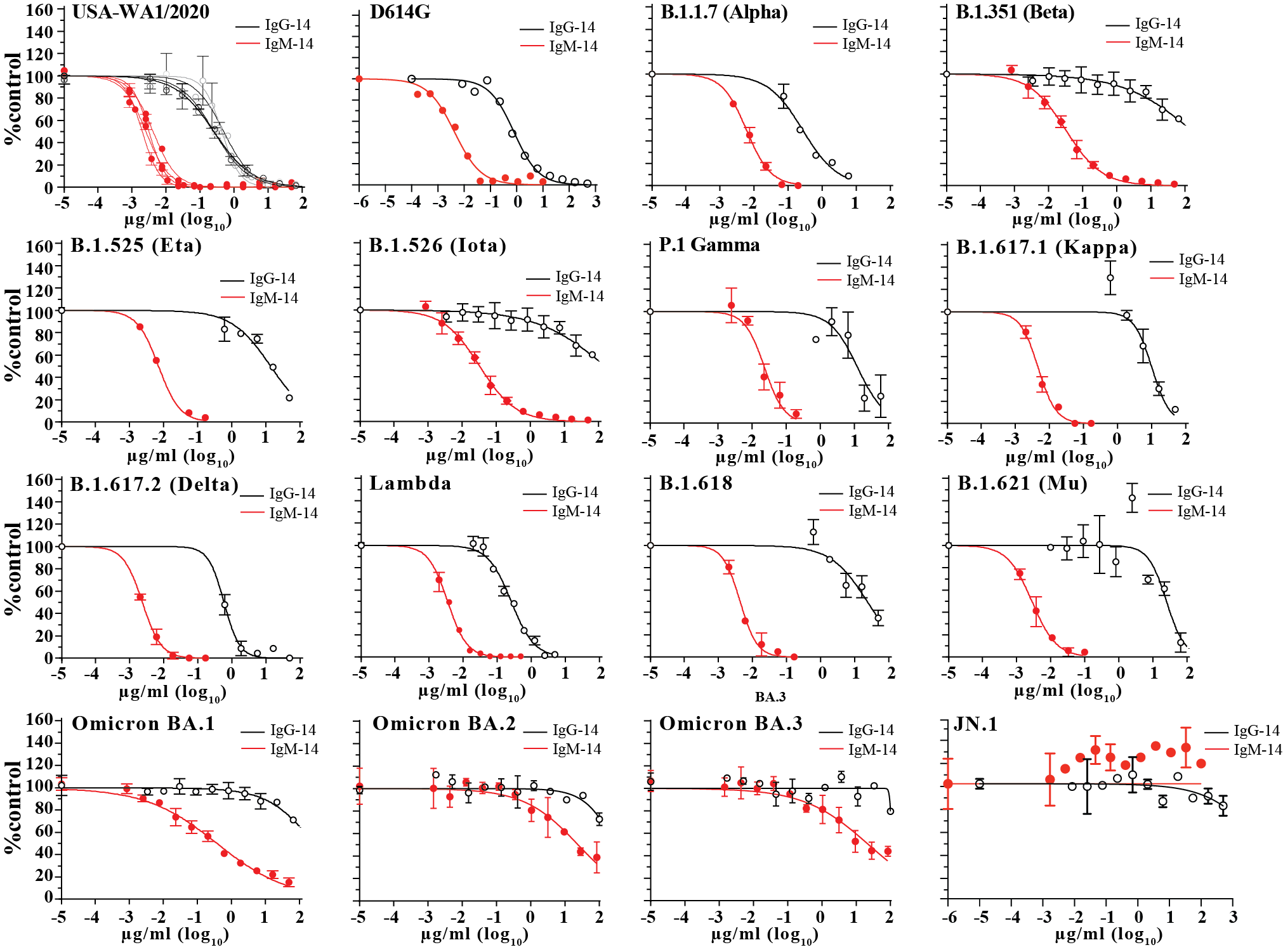


**S1 Fig**. **Neutralization curve of IgM-14 and IgG-14 against SARS-CoV-2 variants**. Red circles and curves represent response to IgM-14 treatment; open black circles with solid dark curves indicate response to IgG-14 treatment. USA-WA1/2020 has been tested for four different batches, each with three replicates. Means from three independent experiments are shown. Error bars indicate standard deviations.
