# Supplementary material for: Neutralization of SARS-CoV-2 by IgM-14 via engagement of two distinct spike epitopes
Source: PLoS Pathog. 2026 Mar 25;22(3):e1014071. doi: 10.1371/journal.ppat.1014071 (PMC13043055; doi:10.1371/journal.ppat.1014071)
Supplement: S2 Fig — A, chromatography of sequences encoding spike amino acids 475–494. B, chromatography of sequences encoding spike amino acids 671–691. Mutations are highlighted. (DOCX) [file ppat.1014071.s002.docx]

**
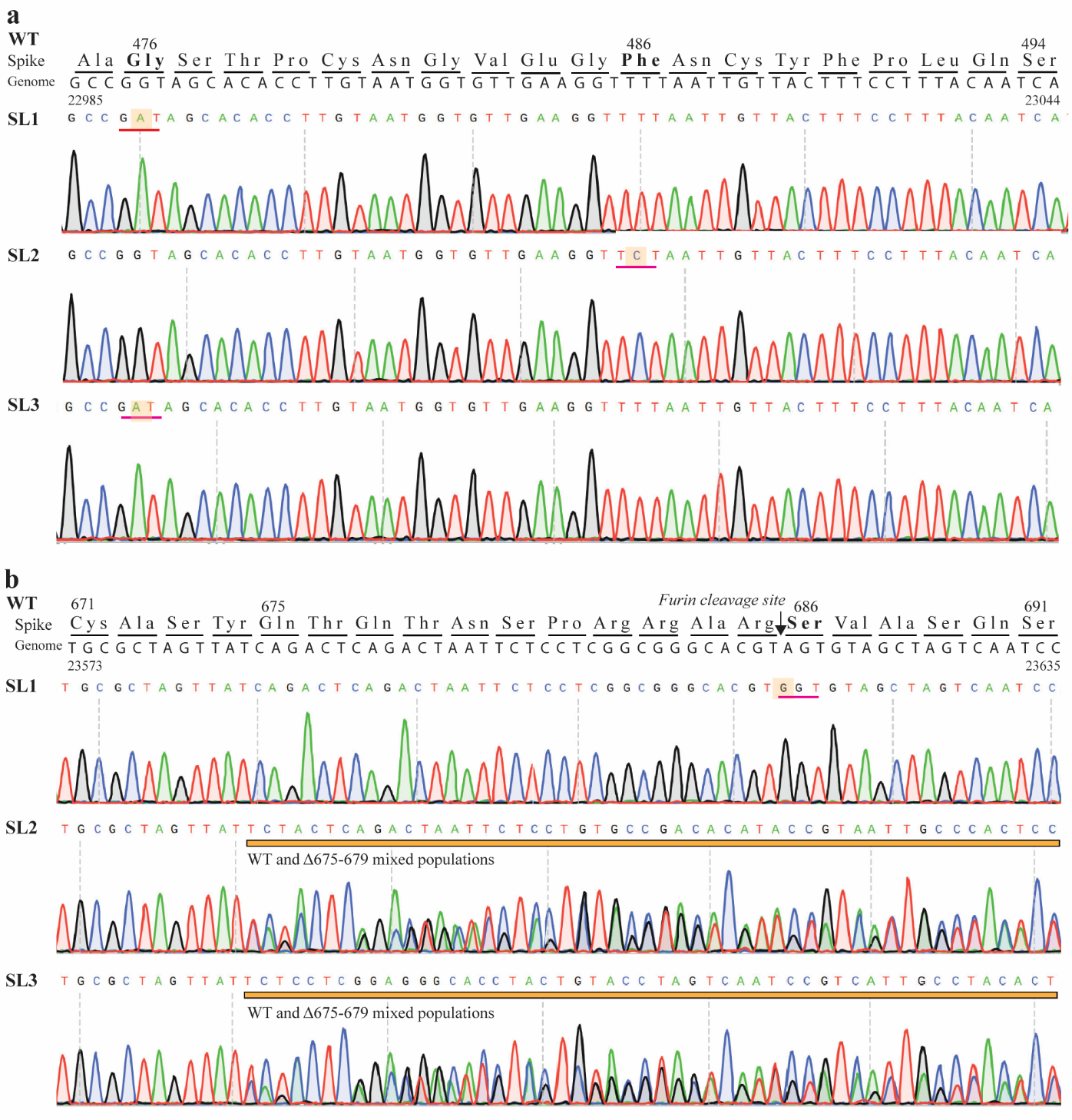
**

**B**

**A**

**S2 Fig**. **Representative Sanger sequencing chromatography of spike gene. A**, chromatography of sequences encoding spike amino acids 475-494. **B**, chromatography of sequences encoding spike amino acids 671-691. Mutations are highlighted.
