# Supplementary material for: Neutralization of SARS-CoV-2 by IgM-14 via engagement of two distinct spike epitopes
Source: PLoS Pathog. 2026 Mar 25;22(3):e1014071. doi: 10.1371/journal.ppat.1014071 (PMC13043055; doi:10.1371/journal.ppat.1014071)
Supplement: S3 Fig — A, SEC analysis of D614G spike mixed with IgM-14. The elution volume for each major peak is indicated. B, SEC analysis of D614G spike mixed with IgG-14. C, Representative negative-stain EM micrographs and 2D class averages of IgM-14 alone. Black circles indicate “starfish-like” IgM-14 particles. D, Representative micrographs and 2D classes of D614G spike alone. Blue circles indicate intact prefusion trimeric spike particles. E, Representative micrographs and 2D classes of D614G spike in the presence of IgM-14 at the indicated molar ratios. Red circles indicate postfusion-like spike particles. Scale bar, 100 nm. (DOCX) [file ppat.1014071.s003.docx]

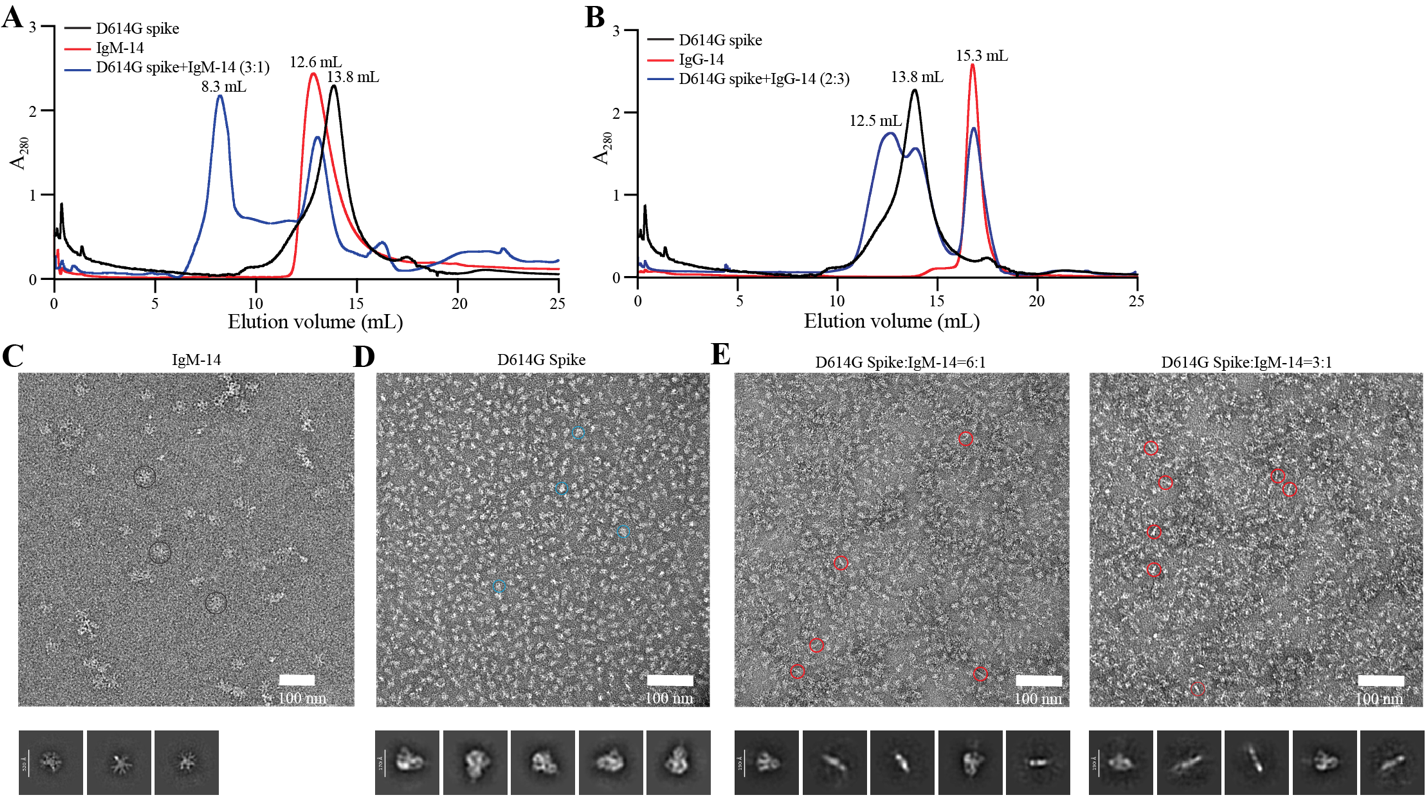


**S3 Fig.** **Size exclusion column and negative-stain EM analysis of D614G spike in complex with IgM-14**. **A**, SEC analysis of D614G spike mixed with IgM-14. The elution volume for each major peak is indicated. **B**, SEC analysis of D614G spike mixed with IgG-14. **C**, Representative negative-stain EM micrographs and 2D class averages of IgM-14 alone. Black circles indicate “starfish-like” IgM-14 particles. **D,** Representative micrographs and 2D classes of D614G spike alone. Blue circles indicate intact prefusion trimeric spike particles. **E,** Representative micrographs and 2D classes of D614G spike in the presence of IgM-14 at the indicated molar ratios. Red circles indicate postfusion-like spike particles. Scale bar, 100 nm.
