# Supplementary material for: Neutralization of SARS-CoV-2 by IgM-14 via engagement of two distinct spike epitopes
Source: PLoS Pathog. 2026 Mar 25;22(3):e1014071. doi: 10.1371/journal.ppat.1014071 (PMC13043055; doi:10.1371/journal.ppat.1014071)
Supplement: S4 Fig — A, Representative cryo-EM micrograph of D614G spike alone. Scale bars, 100 nm. B, Representative 2D classes of the entire dataset. C, Diagram of cryo-EM data process. Two distinct conformations (1-up-RBD and 3-down-RBD) with the corresponding Gold Standard Fourier Shell Correlation (GSFSC) and local resolution estimation are shown. (DOCX) [file ppat.1014071.s004.docx]

**
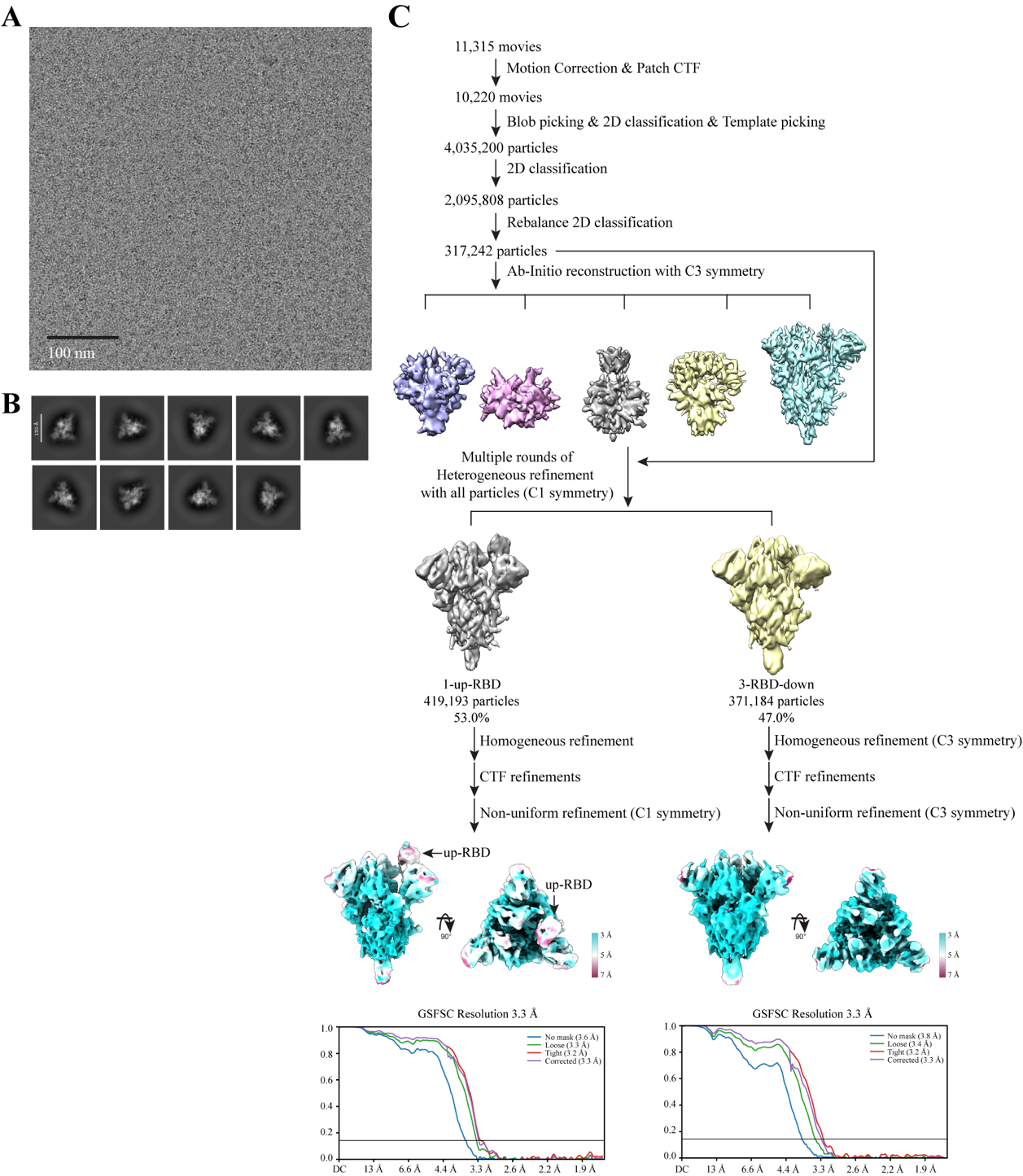
S4 Fig.** **Cryo-EM data processing and reconstruction of D614G spike.** **A,** Representative cryo-EM micrograph of D614G spike alone. Scale bars, 100 nm. **B,** Representative 2D classes of the entire dataset. **C,** Diagram of cryo-EM data process. Two distinct conformations (1-up-RBD and 3-down-RBD) with the corresponding Gold Standard Fourier Shell Correlation (GSFSC) and local resolution estimation are shown.
