# Supplementary material for: Neutralization of SARS-CoV-2 by IgM-14 via engagement of two distinct spike epitopes
Source: PLoS Pathog. 2026 Mar 25;22(3):e1014071. doi: 10.1371/journal.ppat.1014071 (PMC13043055; doi:10.1371/journal.ppat.1014071)
Supplement: S5 Fig — A, Representative micrograph. Scale bars, 100 nm. B, Representative 2D class averages. C, Diagram of cryo-EM data process. Five modes (I-V) were shown. Three subgroups with slightly different up angles in Mode II and two subgroups with slightly different orientations of the Fab-bound RBDs in Mode IV are shown. D, Local refinement of complex Mode I. E, Local refinement of complex Mode IV subgroup I. GSFSC and local resolution estimation for each map are shown. F, Primary binding site from local refinement of Mode I, showing the fitted atomic model within the cryo-EM density. G, Secondary binding site from local refinement of Mode I, showing the fitted atomic model within the cryo-EM density. H, Primary binding site from local refinement of Mode IV, subgroup I, showing the fitted atomic model within the cryo-EM density. (DOCX) [file ppat.1014071.s005.docx]

**
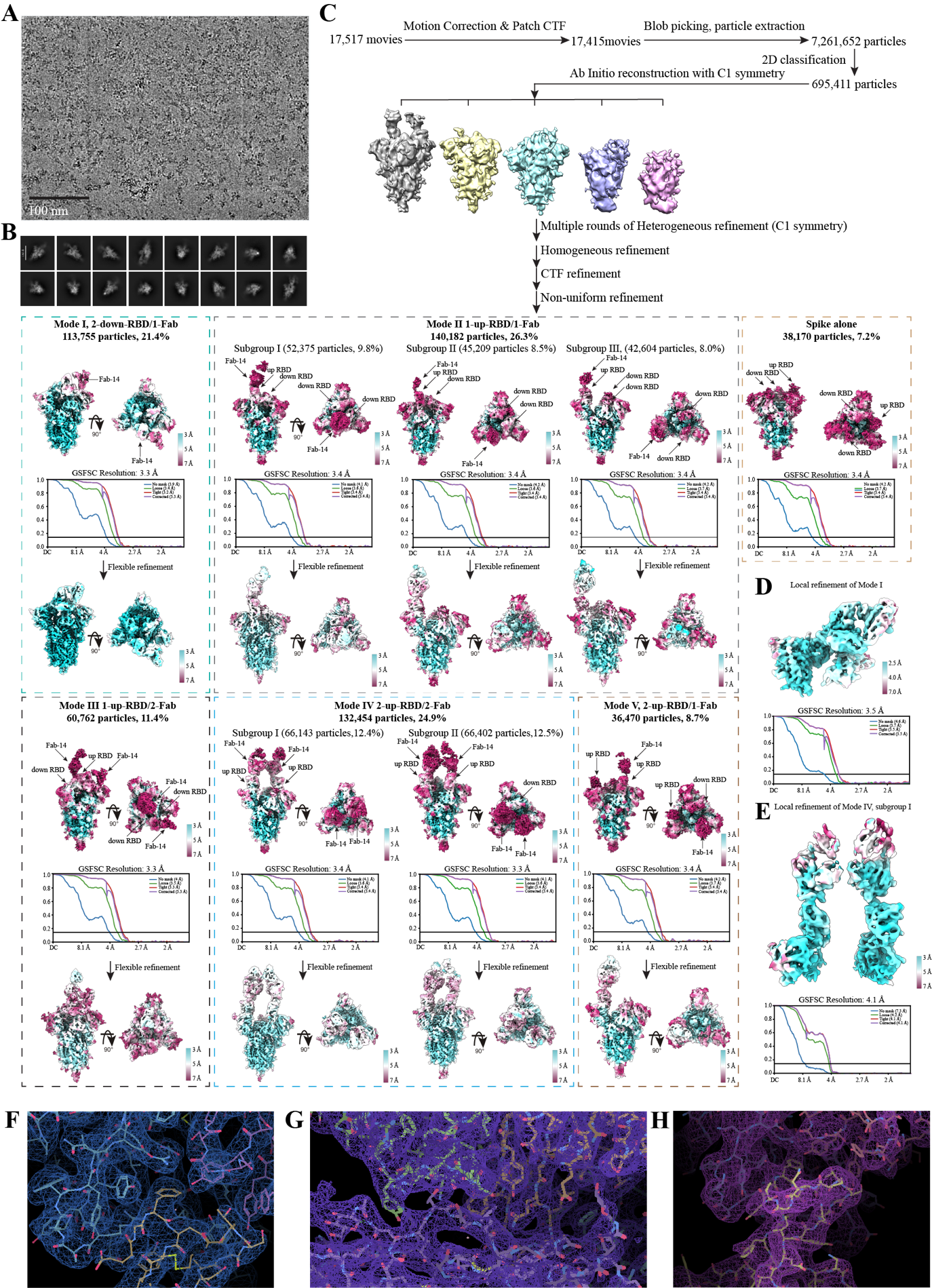
S5 Fig**. **Cryo-EM data processing and reconstruction of D614G spike in complex with Fab-14.** **A,** Representative micrograph. Scale bars, 100 nm. **B,** Representative 2D class averages. **C,** Diagram of cryo-EM data process. Five modes (I-V) were shown. Three subgroups with slightly different up angles in Mode II and two subgroups with slightly different orientations of the Fab-bound RBDs in Mode IV are shown. **D,** Local refinement of complex mode I. **E,** Local refinement of complex mode IV subgroup I. GSFSC and local resolution estimation for each map are shown. **F,** Primary binding site from local refinement of Mode I, showing the fitted atomic model within the cryo-EM density. **G,** Secondary binding site from local refinement of Mode I, showing the fitted atomic model within the cryo-EM density. **H,** Primary binding site from local refinement of Mode IV, subgroup I, showing the fitted atomic model within the cryo-EM density.
