# Supplementary material for: Neutralization of SARS-CoV-2 by IgM-14 via engagement of two distinct spike epitopes
Source: PLoS Pathog. 2026 Mar 25;22(3):e1014071. doi: 10.1371/journal.ppat.1014071 (PMC13043055; doi:10.1371/journal.ppat.1014071)
Supplement: S6 Fig — A, Superimposition of cryo-EM maps for Mode I and its 5 Å low-pass filtered maps. B, Angles between the axis of each RBD in Mode I and the horizontal plane are shown to the right of each chain. C, Distance between each down RBD in Mode I. D, Angles between the axes of RBDs in the D614 spike and the horizontal plane are shown to the right of each chain. E, Distance between the center of mass of each down RBD in D614G spike. D-E, A three-fold symmetric spike structure (PDB ID: 7bnm) was used for comparison. (DOCX) [file ppat.1014071.s006.docx]

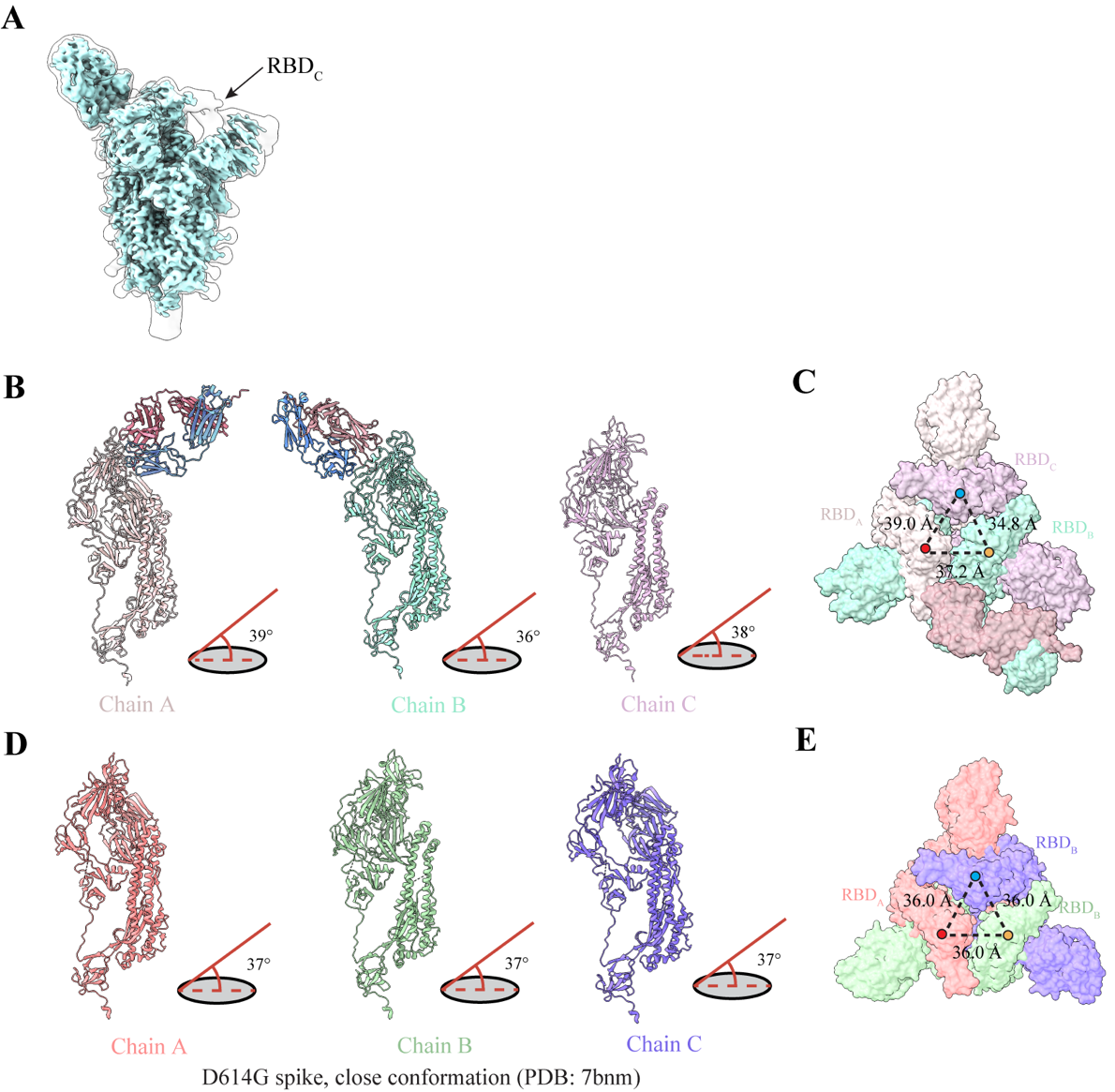


**S6 Fig. Protomer of D614 spike-Fab-14 in Mode I. A**, Superimposition of cryo-EM maps for Mode I and its 5 Å low-pass filtered maps. **B,** Angles between the axis of each RBD in Mode I and the horizontal plane are shown to the right of each chain. **C,** Distance between each down RBD in Mode I. **D,** Angles between the axes of RBDs in the D614 spike and the horizontal plane are shown to the right of each chain. **E,** Distance between the center of mass of each down RBD in D614G spike. **D-E**, A three-fold symmetric spike structure (PDB ID: 7bnm) was used for comparison.
