# Supplementary material for: Neutralization of SARS-CoV-2 by IgM-14 via engagement of two distinct spike epitopes
Source: PLoS Pathog. 2026 Mar 25;22(3):e1014071. doi: 10.1371/journal.ppat.1014071 (PMC13043055; doi:10.1371/journal.ppat.1014071)
Supplement: S7 Fig — A, Alignment of cryo-EM maps of subgroups I-III. B, Angles between the axes of the up RBD (fitted into the cryo-EM maps in rigid bodies) in the three subgroups. (DOCX) [file ppat.1014071.s007.docx]

**
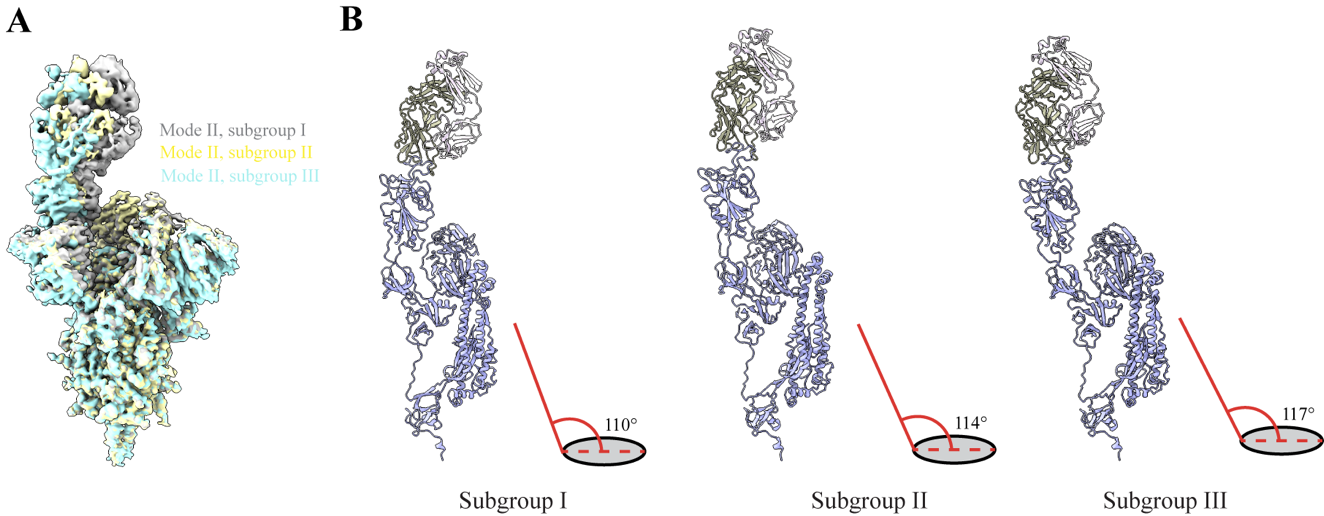
S7 Fig. Analysis of up RBD in three subgroups of Mode II.** **A,** Alignment of cryo-EM maps of subgroups I-III. **B,** Angles between the axes of the up RBD (fitted into the cryo-EM maps in rigid bodies) in the three subgroups.
