# Supplementary material for: Neutralization of SARS-CoV-2 by IgM-14 via engagement of two distinct spike epitopes
Source: PLoS Pathog. 2026 Mar 25;22(3):e1014071. doi: 10.1371/journal.ppat.1014071 (PMC13043055; doi:10.1371/journal.ppat.1014071)

**S8 Fig. Analysis of Mode III.** **A,** Angles between the axes of each RBD (fitted into the cryo-EM maps in rigid bodies) in three subgroups. **B,** Top view of Mode III. Distance between the centers of mass of two down RBDs.


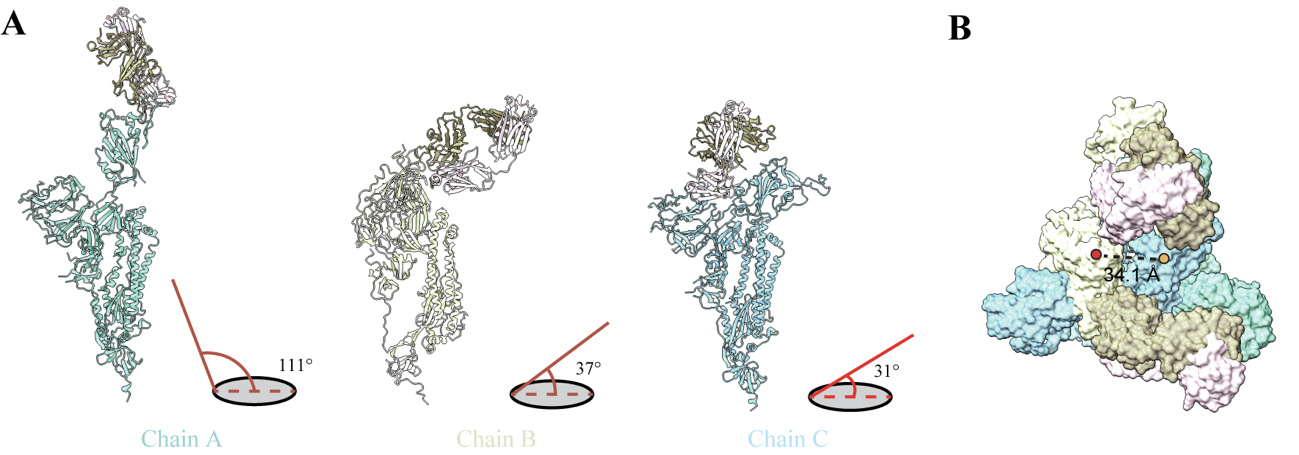

Supplement: S8 Fig — A, Angles between the axes of each RBD (fitted into the cryo-EM maps in rigid bodies) in three subgroups. B, Top view of Mode III. Distance between the centers of mass of two down RBDs. (DOCX) [file ppat.1014071.s008.docx]
