# Supplementary material for: Neutralization of SARS-CoV-2 by IgM-14 via engagement of two distinct spike epitopes
Source: PLoS Pathog. 2026 Mar 25;22(3):e1014071. doi: 10.1371/journal.ppat.1014071 (PMC13043055; doi:10.1371/journal.ppat.1014071)
Supplement: S9 Fig — A, Angles between the axes of each up RBD in Mode IV subgroup I. B, Angles between the axes of each up RBD in Mode IV subgroup II. C, Side view of the two up RBDs in Mode IV subgroup I and Mode IV subgroup II. The dashed line indicates the center distance between RBDs. D, Superimposition of cryo-EM maps for Mode V and its 10 Å low-pass filtered maps. E, Scatter plots illustrating the dispersion of particle latent coordinates throughout the Mode V dataset. F, A representative cryo-EM map of Mode V after 3D flexible refinement training. (DOCX) [file ppat.1014071.s009.docx]

**
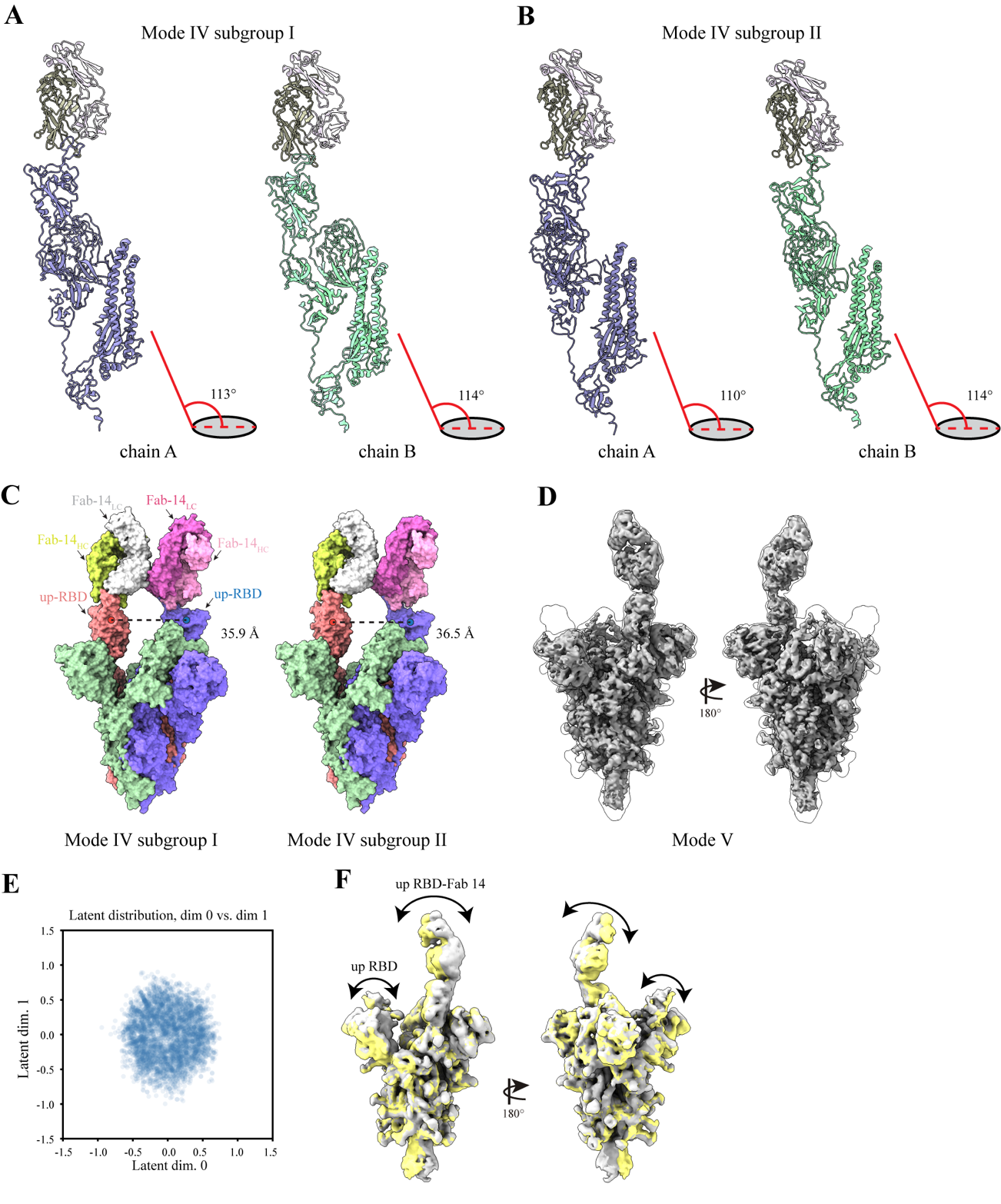
S9 Fig. Analysis of Mode IV and Mode V.** **A,** Angles between the axes of each up RBD in Mode IV subgroup I. **B,** Angles between the axes of each up RBD in Mode IV subgroup II. **C,** Side view of the two up RBDs in Mode IV subgroup I and Mode IV subgroup II. The dashed line indicates the center distance between RBDs. **D,** Superimposition of cryo-EM maps for Mode V and its 10 Å low-pass filtered maps. **E,** Scatter plots illustrating the dispersion of particle latent coordinates throughout the Mode V dataset. **F,** A representative cryo-EM map of mode V after 3D flexible refinement training.
