# Supplementary material for: Neutralization of SARS-CoV-2 by IgM-14 via engagement of two distinct spike epitopes
Source: PLoS Pathog. 2026 Mar 25;22(3):e1014071. doi: 10.1371/journal.ppat.1014071 (PMC13043055; doi:10.1371/journal.ppat.1014071)
Supplement: S10 Fig — A, Structural comparison of two up RBD-Fab-14 after local refinement. B, Interactions between two Fab-14s. C, Structural comparison of the primary binding site when Fab-14 is bound to a down-RBD and an up-RBD. The Fab-14 complexed with down-RBD is shown in gray, while Fab-14 complexed with up-RBD is shown in salmon. (DOCX) [file ppat.1014071.s010.docx]

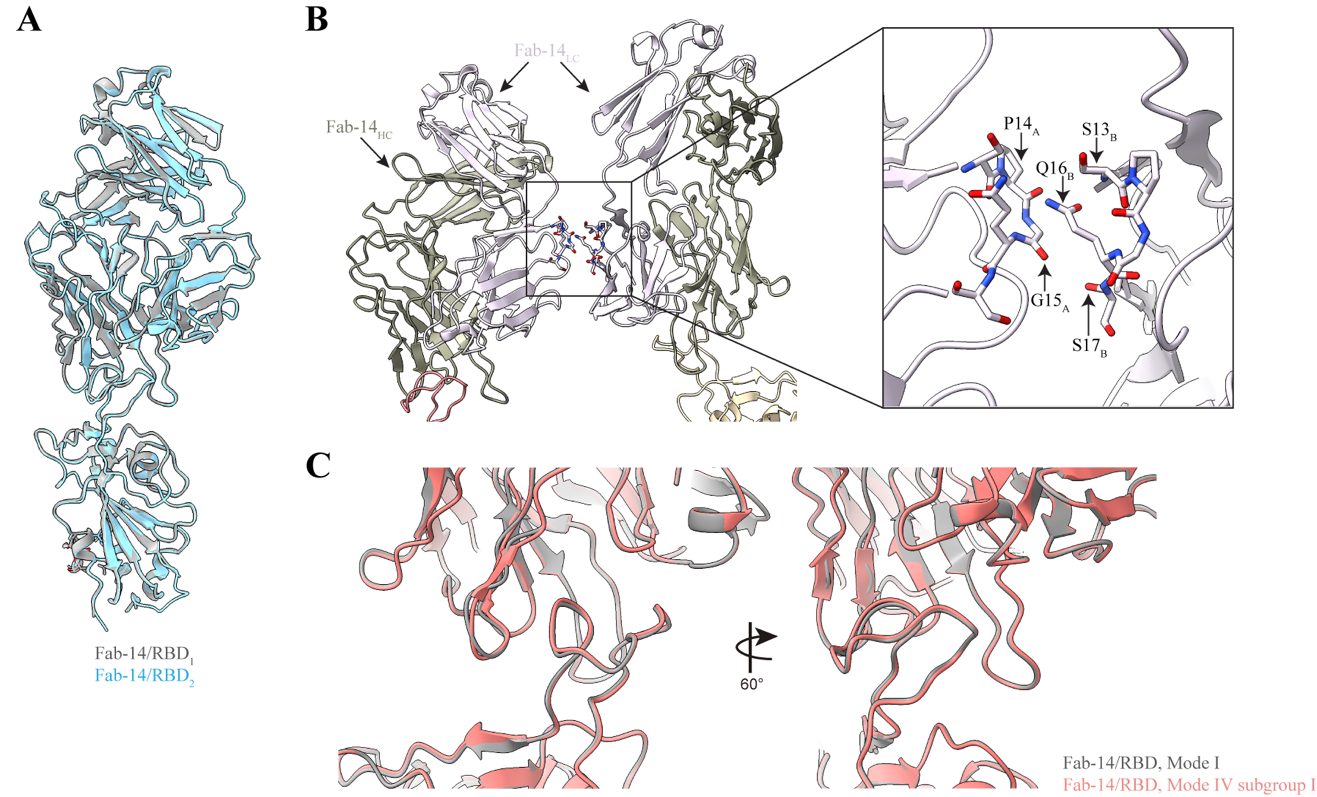


**S10 Fig. Structural analysis of the primary binding site.** **A,** Structural comparison of two up RBD-Fab-14 after local refinement. **B,** Interactions between two Fab-14s. **C,** Structural comparison of the primary binding site when Fab-14 is bound to a down-RBD and an up-RBD. The Fab-14 complexed with down-RBD is shown in gray, while Fab-14 complexed with up-RBD is shown in salmon.
