# Supplementary material for: Neutralization of SARS-CoV-2 by IgM-14 via engagement of two distinct spike epitopes
Source: PLoS Pathog. 2026 Mar 25;22(3):e1014071. doi: 10.1371/journal.ppat.1014071 (PMC13043055; doi:10.1371/journal.ppat.1014071)
Supplement: S11 Fig — RBD was immobilized on the sensor at 5 (top), 20 (middle), or 100 (bottom) µg/ml and titrated with Fab-14 at the indicated concentrations. Apparent kinetic parameters (KD, kon, and koff) and fitting R2 (1:1 binding) values derived from global fitting are indicated. (DOCX) [file ppat.1014071.s011.docx]

**
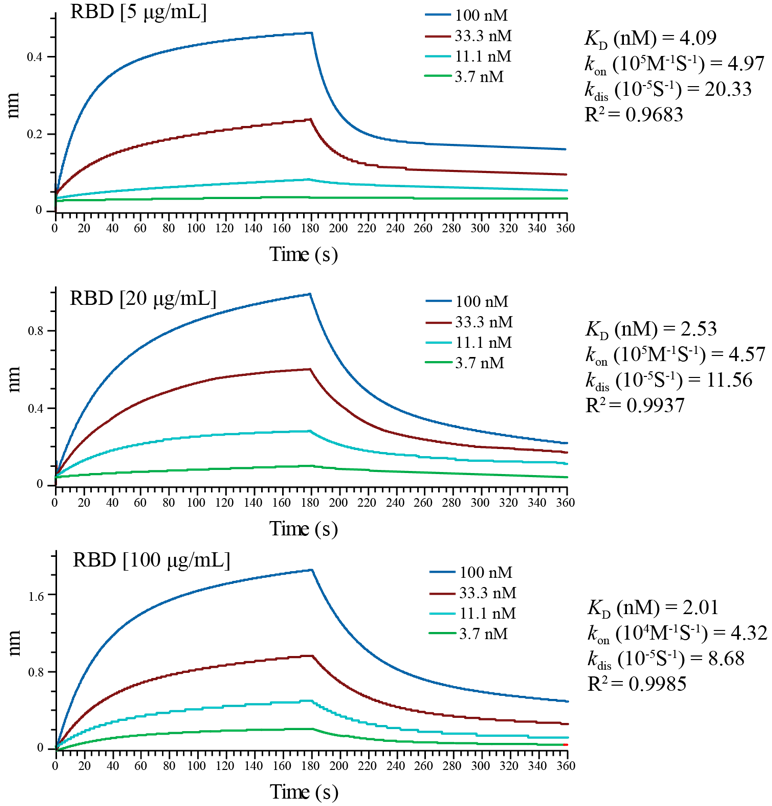
S11 Fig. BLI analysis of Fab-14 binding to immobilized RBD at different surface densities.** RBD was immobilized on the sensor at 5 (top), 20 (middle), or 100 (bottom) µg/mL and titrated with Fab-14 at the indicated concentrations. Apparent kinetic parameters (*K*_D_, *k*_on_, and *k*_off_) and fitting R^2^ (1:1 binding) values derived from global fitting are indicated.
