# Supplementary material for: Neutralization of SARS-CoV-2 by IgM-14 via engagement of two distinct spike epitopes
Source: PLoS Pathog. 2026 Mar 25;22(3):e1014071. doi: 10.1371/journal.ppat.1014071 (PMC13043055; doi:10.1371/journal.ppat.1014071)
Supplement: S12 Fig — A, Representative micrograph. Scale bars, 100 nm. B, Representative 2D classes. C, Diagram of cryo-EM data process. Three distinct conformations were resolved. Confirmation 1–2 represents Fab-free BA.1 spike protein, where conformation 1 contains one up-RBD and conformation 2 has three down-RBD. Confirmation 3 has one Fab-14 bind to two down-RBD. The GSFSC and local resolution estimation for each map were shown. (DOCX) [file ppat.1014071.s012.docx]

**
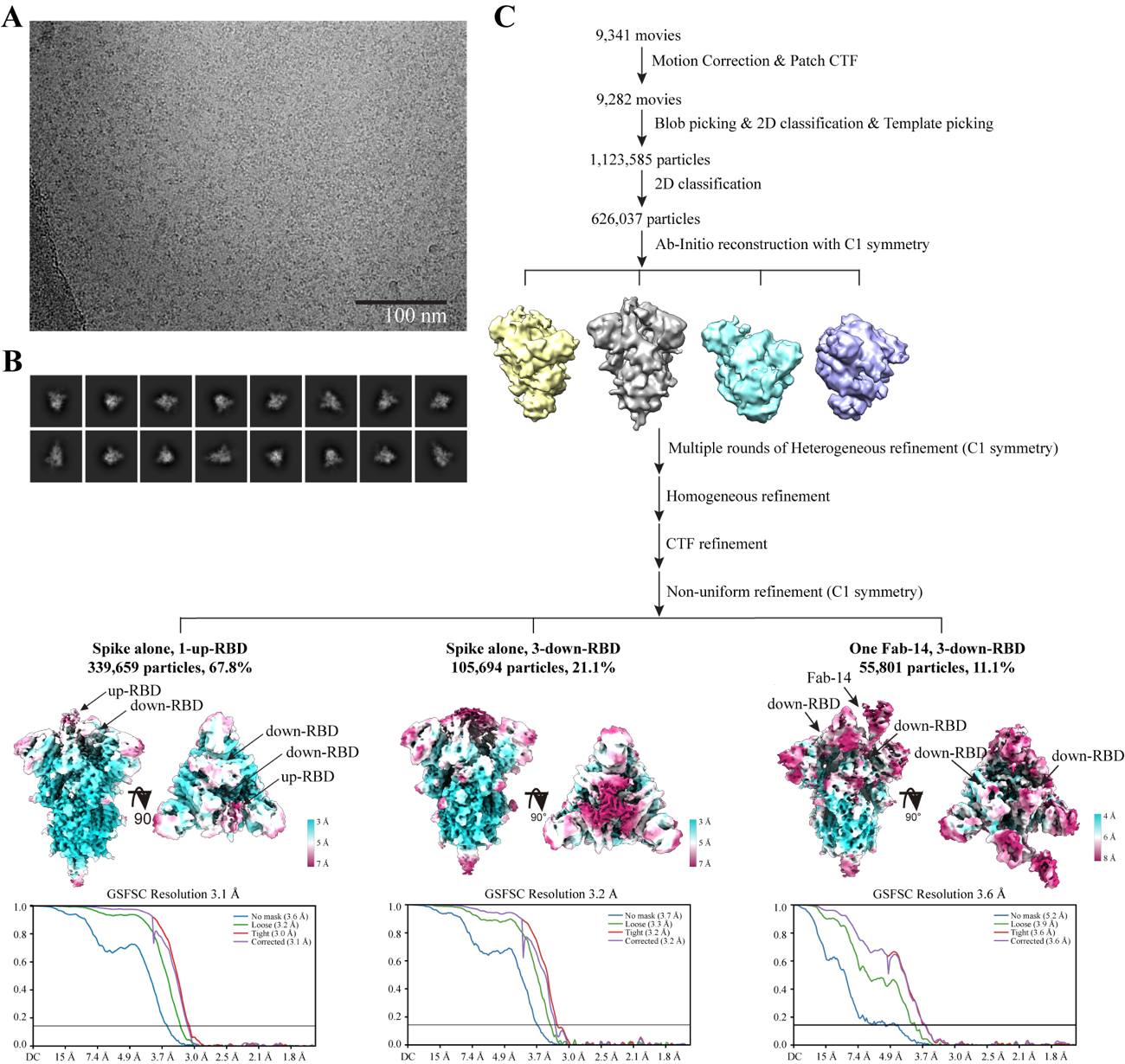
S12 Fig.** **Cryo-EM processing and validation of Omicron BA.1 Spike and Fab-14 complex.** **A,** Representative micrograph. Scale bars, 100 nm. **B,** Representative 2D classes. **C,** Diagram of cryo-EM data process. Three distinct conformations were resolved. Confirmation 1-2 represents Fab-free BA.1 spike protein, where conformation 1 contains one up-RBD and conformation 2 has three down-RBD. Confirmation 3 has one Fab-14 bind to two down-RBD. The GSFSC and local resolution estimation for each map were shown.
