# Supplementary material for: Neutralization of SARS-CoV-2 by IgM-14 via engagement of two distinct spike epitopes
Source: PLoS Pathog. 2026 Mar 25;22(3):e1014071. doi: 10.1371/journal.ppat.1014071 (PMC13043055; doi:10.1371/journal.ppat.1014071)
Supplement: S13 Fig — The down RBD at the primary site is shown in yellow. The down RBD at the secondary site is shown in dark blue. (DOCX) [file ppat.1014071.s013.docx]

**
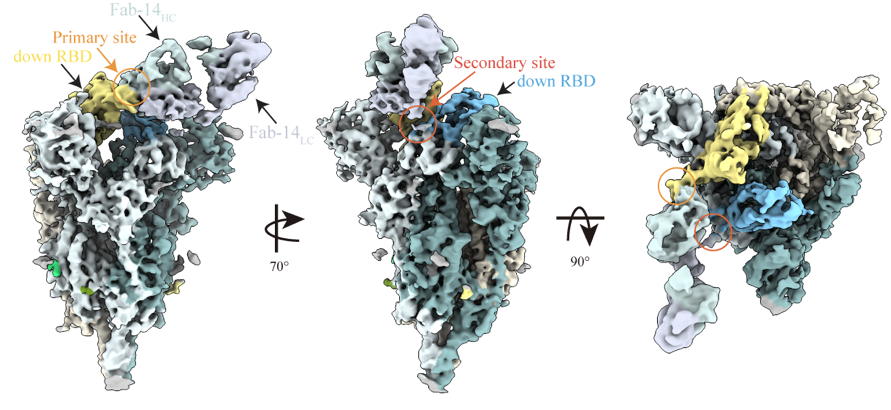
**

**S13 Fig.** **Cryo-EM density map for the Fab-14/BA.1 spike complex.** The down RBD at the primary site is shown in yellow. The down RBD at the secondary site is shown in dark blue.
