# Supplementary material for: Neutralization of SARS-CoV-2 by IgM-14 via engagement of two distinct spike epitopes
Source: PLoS Pathog. 2026 Mar 25;22(3):e1014071. doi: 10.1371/journal.ppat.1014071 (PMC13043055; doi:10.1371/journal.ppat.1014071)
Supplement: S1 Table — (DOCX) [file ppat.1014071.s014.docx]

**S1 Table. Spike mutations in SARS-CoV-2 variants.**

| SARS-CoV-2 variants | Spike mutations | | | |
| --- | --- | --- | --- | --- |
|  | S1 | | | S2 |
|  | NTD | RBD | CTD |  |
| USA-WA1/2020 | Reference | | | |
| D614G | - | - | D614G | - |
| B.1.1.7 (Alpha) | Δ69-70, Δ145, | N501Y | A570D, D614G, P681H | T716I, S982A, D1118H |
| B.1.351 (Beta) | D80A, D215G, Δ242-244 | K417N, E484K, N501Y | D614G | A701V |
| B.1.525 (Eta) | Q52R, A67V, Δ69-70, Δ145 | E484K | D614G, Q677H | F888L |
| B.1.526 (Iota) | L5F, T95I, D253G | E484K | D614G | A701V |
| P.1 (Gamma) | L18F, T20N, P26S, D138Y, R190S | K417T, E484K, N501Y | D614G, H655Y | T1027I, V1176F |
| B.1.617.1 (Kappa) | G142D, E154K | L452R, E484Q | D614G, P681R | Q1071H, H1101D |
| B.1.617.2 (Delta) | T19R, G142D, E156G, Δ157-158, | L452R, T478K | D614G, P681R | D950N |
| C.37 (Lambda) | G75V, T76I, Δ246-252, D253N | L452Q, F490S | D614G | T859N |
| B.1.618 | H49Y, Δ145-146, | E484K | D614G | - |
| B.1.621 (Mu) | T19R, T95I, Ins143T, Y144S, Y145N | R346K, E484K, N501Y | D614G, P681H | D950N |
| Omicron sublineage BA.1 | A67V, Δ69-70, T95I, G142D, Δ143-145, Δ211, L212I, Ins214EPE | G339D, S371L, S373P, S375F, K417N, N440K, G446S, S477N, T478K, E484A, Q493R, G496S, Q498R, N501Y, Y505H | T547K, D614G, H655Y, N679K, P681H | N764K, D796Y, N856K, Q954H, N969K, L981F |
| Omicron sublineage BA.2 | T19I, Δ24-26, A27S, G142D, V213G | G339D, S371F, S373P, S375F, T376A, D405N, R408S, K417N, N440K, S477N, T478K, E484A, Q493R, Q498R, N501Y, Y505H | D614G, H655Y, N679K, P681H | N764K, D796Y, Q954H, N969K |
| Omicron sublineage BA.3 | A67V, Δ69-70, T95I, G142D, Δ143-145, Δ211, L212I | G339D, S371L, S373P, S375F, D405N, K417N, N440K, G446S, S477N, T478K, E484A, Q493R, Q498R, N501Y, Y505H | D614G, H655Y, N679K, P681H | N764K, D796Y, Q954H, N969K |
| Omicron sublineage JN.1 | Ins16MPLF, T19I, R21T, Δ24-26, A27S, S50L, Δ69-70, V127F,G142D, Δ145, F157S, R158G, Δ211, L212I, V213G, L216F, H245N, A264D | I332V, G339H, K356T, S371F, S373P, S375F, T376A, R403K, D405N, R408S, K417N, N440K, V445H, G446S, N450D, L452W, L455S, N460K, S477N, T478K, N481K, Δ483, E484K, F486P, Q498R, N501Y | Y550H, E554K, A570V, D614G, P621S, H655Y, I670V, N679K, P681R | N764K, D796Y, S939F, Q954H, N969K, P1143L |
