# Supplementary material for: Neutralization of SARS-CoV-2 by IgM-14 via engagement of two distinct spike epitopes
Source: PLoS Pathog. 2026 Mar 25;22(3):e1014071. doi: 10.1371/journal.ppat.1014071 (PMC13043055; doi:10.1371/journal.ppat.1014071)
Supplement: S2 Table — (DOCX) [file ppat.1014071.s015.docx]

**S2 Table. List of primers used in this study.**

| Primer Name | Sequence (5’-3’) | Notes |
| --- | --- | --- |
| G476D-F | gaaatctatcaggccgAtagcacaccttg | For construction mNG SARS-COV-2 spike mutation G476D |
| G476D-R | caaggtgtgctaTcggcctgatagatttc |  |
| F486S-F | ggtgttgaaggttCtaattgttactttc | For construction mNG SARS-COV-2 spike mutation F486S |
| F486S-R | gaaagtaacaattaGaaccttcaacacc |  |
| E484A-F | CCTTGTAATGGTGTTGCAGGTTTTAATTGTTACTTTCC | For construction mNG SARS-COV-2 spike mutation E484A |
| E484A-R: | TAACAATTAAAACCGTCAACACCATTACAAGGTGTGC |  |
| L335A+N331Q-F | GATTTCCTCAGATTACAAACGCGTGCCCTTTTGGTGAAG | For construction mNG SARS-COV-2 spike mutation L335A and N331Q |
| L335A+N331Q-R | CAAAAGGGCACGCGTTTGTAATACGAGGAAATCTAACAATAG |  |
| Cov-21115V | CATTTGTGGGTTTATACAACAAAAG | For amplifying cDNAs by RT-PCR for sequencing |
| Cov-25169R | GTTCTTGGAGATCGATGAGAGATTC |  |
