# Supplementary material for: Neutralization of SARS-CoV-2 by IgM-14 via engagement of two distinct spike epitopes
Source: PLoS Pathog. 2026 Mar 25;22(3):e1014071. doi: 10.1371/journal.ppat.1014071 (PMC13043055; doi:10.1371/journal.ppat.1014071)
Supplement: S3 Table — (DOCX) [file ppat.1014071.s016.docx]

**S3 Table**. **Statistics for 3D reconstruction for D614G spike alone.**

|  | 1-RBD-up | 3-RBD-down | |
| --- | --- | --- | --- |
| EMD | 73231 | 73244 | |
| **Data collection and processing** | | | |
| Microscope | Krios | | |
| Camera | Falcon 4 | | |
| Voltage (keV) | 300 | | |
| Defocus range () | 0.86 | | |
| Pixel size (Å) | -1.0 to -2.5 | | |
| Electron dose (^−^ Å^−1^) | 40 | | |
| **Refinement** | | | |
| Symmetry imposed | C3 | C1 | |
| Particles (no.) | 419,193 | 371,184 | |
| Map resolution | 3.3 | 3.3 | |
| Micrographs (no.) | 10,220 | | 10,220 |
