# Supplementary material for: Neutralization of SARS-CoV-2 by IgM-14 via engagement of two distinct spike epitopes
Source: PLoS Pathog. 2026 Mar 25;22(3):e1014071. doi: 10.1371/journal.ppat.1014071 (PMC13043055; doi:10.1371/journal.ppat.1014071)
Supplement: S4 Table — (DOCX) [file ppat.1014071.s017.docx]

**S4 Table**. **Statistics for 3D reconstruction and model refinement of Fab-14/D614G spike complex.**

|  | Mode I | Mode II | | | Mode III | Mode IV | | Mode V | Spike alone |
| --- | --- | --- | --- | --- | --- | --- | --- | --- | --- |
|  |  | Subgroup I | Subgroup II | Subgroup III |  | Subgroup I | Subgroup II |  |  |
| EMD | 73260 | 73263 | 73265 | 73267 | 73290 | 73306 | 73292 | 73247 | 73291 |
| PDB | 9YOK | - | - | - | - | 9YPR | 9YPB | - | - |
| **Data collection and processing** | | | | | | | | | |
| Microscope | Krios | | | | | | | | |
| Camera | K3 | | | | | | | | |
| Voltage (keV) | 300 | | | | | | | | |
| Defocus range (- μm) | 1.0-2.5 | | | | | | | | |
| Pixel size (Å) | 0.84 | | | | | | | | |
| Electron dose (^−^ Å^−1^) | 43.17 | | | | | | | | |
| **Refinement** | | | | | | | | | |
| Symmetry imposed | C1 | C1 | C1 | C1 | C1 | C1 | C1 | C1 | C1 |
| Particles (no.) | 113,755 | 52,375 | 45,209 | 42,604 | 60,762 | 66,143 | 66,402 | 46,470 | 38,170 |
| Map resolution (Å) | 3.3 | 3.4 | 3.4 | 3.4 | 3.3 | 3.3 | 3.4 | 3.3 | 3.4 |
| Micrographs (no.) | 17,415 | 17,415 | 17,415 | 17,415 | 17,415 | 17,415 | 17,415 | 17,415 | 17,415 |
| **Composition** |  | | | | | | | | |
| Chains | 5 | - | - | - | - | 7 | 7 | - | - |
| Atoms | 27,067 | - | - | - | - | 27,948 | 29,861 | - | - |
| Residues (Protein) | 3,409 | - | - | - | - | 3,549 | 3,804 | - | - |
| Ligands | 38 | - | - | - | - | 31 | 25 | - | - |
| **R.m.s. deviations** | | | | | | | | | |
| Bond length (Å) | 0.004 | - | - | - | - | 0.004 | 0.005 | - | - |
| Bond angles (°) | 0.604 | - | - | - | - | 0.691 | 1.011 | - | - |
| **Model statistics** | | | | | | | | | |
| Clash score | 11.83 | - | - | - | - | 13.46 | 15.93 | - | - |
| MolProbity score | 2.01 | - | - | - | - | 2.05 | 2.12 | - | - |
| Rotamer outliers (%) | 0.71 | - | - | - | - | 0.84 | 0.94 | - | - |
| **Ramachandran plot** | | | | | | | | | |
| Outliers (%) | 0.00 | - | - | - | - | 0.03 | 0.03 | - | - |
| Allowed (%) | 6.52 | - | - |  | - | 6.07 | 6.17 | - | - |
| Favored (%) | 93.48 | - | - | - | - | 93.90 | 93.80 | - | - |
