# Supplementary material for: Neutralization of SARS-CoV-2 by IgM-14 via engagement of two distinct spike epitopes
Source: PLoS Pathog. 2026 Mar 25;22(3):e1014071. doi: 10.1371/journal.ppat.1014071 (PMC13043055; doi:10.1371/journal.ppat.1014071)
Supplement: S5 Table — (DOCX) [file ppat.1014071.s018.docx]

**S5 Table. Statistics for 3D reconstruction and local refinement of Fab-14/D614G RBD structure.**

|  | 2-down-RBD/1-Fab | 2-up-RBD/2-Fab |
| --- | --- | --- |
| EMD | 73228 | 73245 |
| PDB | 9YNR | 9YNX |
| **Refinement** | | |
| Symmetry imposed | C1 | C1 |
| Particles (no.) | 113,755 | 66,402 |
| Map resolution (Å) | 3.5 | 4.1 |
| **Composition** | | |
| Chains | 4 | 4 |
| Atoms | 9,679 | 9,767 |
| Residues (Protein) | 1,217 | 1,266 |
| Ligands | 13 | 4 |
| **R.m.s. deviations** | | |
| Bond length (Å) | 0.005 | 0.004 |
| Bond angles (°) | 0.649 | 0.671 |
| **Model statistics** | | |
| Clash score | 7.20 | 11.33 |
| MolProbity score | 1.78 | 1.95 |
| Rotamer outliers (%) | 0.94 | 0.00 |
| **Ramachandran plot** | | |
| Outliers (%) | 0.00 | 0.00 |
| Allowed (%) | 5.71 | 5.51 |
| Favored (%) | 94.29 | 94.49 |
