# Supplementary material for: Neutralization of SARS-CoV-2 by IgM-14 via engagement of two distinct spike epitopes
Source: PLoS Pathog. 2026 Mar 25;22(3):e1014071. doi: 10.1371/journal.ppat.1014071 (PMC13043055; doi:10.1371/journal.ppat.1014071)
Supplement: S6 Table — (DOCX) [file ppat.1014071.s019.docx]

**S6 Table. Statistics for 3D reconstruction of Fab-14/BA.1 spike complex.**

|  | Spike alone, 1-RBD-up | Spike alone, 3- RBD-down | Spike/Fab-14 complex, 3-RBD-down |  |
| --- | --- | --- | --- | --- |
| EMD | 73273 | 73271 | 73270 |  |
| **Data collection and processing** | | | | |
| Microscope | Krios | | | |
| Camera | K3 | | | |
| Voltage (keV) | 300 | | | |
| Defocus range (- μm) | 1.0-2.5 | | | |
| Pixel size | 0.84 | | | |
| Electron dose (^−^ Å^−1^) | 43.27 | | | |
| **Refinement** | | | | |
| Symmetry imposed | C1 | C3 | C1 |  |
| Particles (no.) | 339,659 | 105,694 | 55,801 |  |
| Map resolution | 3.1 | 3.2 | 3.6 |  |
| Micrographs (no.) | 9,282 | 9,282 | 9,282 |  |
