# Supplementary material for: Neutralization of SARS-CoV-2 by IgM-14 via engagement of two distinct spike epitopes
Source: PLoS Pathog. 2026 Mar 25;22(3):e1014071. doi: 10.1371/journal.ppat.1014071 (PMC13043055; doi:10.1371/journal.ppat.1014071)
Supplement: Attachment — Submitted filename: Response to Reviewers.docx [file ppat.1014071.s022.docx]

**Part I – Summary**

Reviewer #1: In this manuscript, Wang et al. investigate the neutralization activity of a previously characterized SARS-CoV-2 IgM antibody, IgM-14, against newly emerged variants and present cryo-EM structures of Fab-Spike complexes. By extending earlier functional observations to additional variants and providing structural information, the manuscript offers useful insights into the neutralizing mechanism of the IgM-14. The study is technically sound, the data are of good quality, and the overall scope and depth of the work are well aligned with the standards of the journal. Providing more solid evidence and explanations in the following aspects would further strengthen the manuscript.

Reviewer #2: This study examines IgM-14, an engineered IgM antibody against SARS-CoV-2, and explains why it is so potent and how viral variants escape it. They found IgM-14 is far more powerful than its IgG version against all pre-Omicron SARS-CoV-2 variants, but its activity drops sharply against Omicron BA.1 and is completely lost against the later JN.1 variant. Two mutations on Spike RBD, G476D and F486P, were found with resistance to the IgM-14. Further cryo-EM structural study revealed two binding interfaces on RBD, which explains why the IgM-14 showed enhanced potency than IgG-14. Overall, the study is important and interesting, providing a new dual-engage model of antibody neutralization.

Reviewer #3: In the manuscript titled “Neutralization of SARS-CoV-2 by IgM-14 via Engagement of Two Distinct Spike Epitopes,” Wang, Hu et al. study the mechanism of SARS-CoV-2 neutralization by IgM-14, a therapeutic antibody that was administered intranasally. The main finding of the paper is that the IgM version of the antibody leverages the secondary contact that the Fab makes with the adjacent RBD. By determining cryo-EM structures of Fab-14 with spike, the authors have revealed high-resolution details of the interaction. Overall, the paper reports important data on a clinically relevant antibody and provide a model for multivalent association with IgM.

**Response:** We appreciate all reviewers for their positive and constructive feedback.

The authors should address the following:

1. The NSEM data are not sufficient to draw the conclusion that IgM-14 promoted aggregation. The spike sample itself shows some clustering and the IgM+spike sample looks messier but still to make the conclusion about IgM-mediated aggregation, the authors should use a complementary measure such as light scattering or size exclusion chromatography.

**Response**: We thank the reviewer for this excellent suggestion. We performed size-exclusion chromatography (SEC) to analyze IgM-14/spike samples. D614 spike was incubated with IgM at a molar ratio of 3:1 (approximately 1:1 RBD: Fab-14) and analyzed by SEC using a Superpose 6 Increase 10/300 column, with IgG-14 included as a control under identical conditions. Both SEC and NSEM results suggest IgM14’s capability in promoting spike-cross-linking/aggregation. The new SEC data have been added to the revised manuscript (Lines 190-202 and Fig S3A-B).

2. The use of the MM/PBSA calculations seems underdeveloped and are not adding more insights beyond what can be observed directly with the structures. These calculations do not consider the impact of the mutations on the conformational landscape of the spike and are an oversimplification, therefore, likely to be misleading. I recommend removing this and focusing solely on the experimental structural and functional analysis.

**Response**: We thank the reviewer for this good point. We agree with the reviewer that there is a limitation for the MM/PBSA analysis. Despite overall consistency between MM/PBSA and our experimental observation, we have carefully rephrased the MM/PBSA results with emphasis on experimental structural and functional analysis to avoid misleading (Lines 290-296 and 311-312).

3. In the supplement, authors should show overlays of the map and model with the map contoured appropriately to demonstrate resolution. This should be done for different regions of the structures, especially the Fab/spike interactive regions.

**Response**: We thank the reviewer for this good point. Map-model overlays of the Fab-14 and RBD interface, including both primary and secondary binding sites, have been added as Fig. S5F-H in the revised manuscript.

4. In the section covered in lines 279-287, the authors refer to distances and hydrogen bonds and main chain interactions, but none of these are shown in figure (5A). Authors should revise the figure.

**Response**: Corrected. Figure 5A has been updated to show the hydrogen networks.

5. Line 347-348 – Other antibodies are known that make peripheral contact with the adjacent RBD or the adjacent NTD. What this paper shows that these secondary contacts can be functionally relevant. Authors should consider revising/modulating this statement.

**Response**: Corrected. This statement has been rephrased as below in the revised manuscript. “This bipartite binding mode appears to be unique to Fab-14; to our knowledge, this is the first report demonstrating the functional relevance of such a configuration in the IgM” (Line 372-373).

6. For the BLI data, authors should show the fit to the curves overlayed on the sensorgrams. For some of the interactions (for example, 2J WT), there is almost nothing falling off the sensor tip in the off phase, therefore, how accurate/robust are the affinity and kinetics values provided?

**Response**: We included the full R² values from the curve fitting in Figure 2J. The raw fitting results are shown as bellow. Despite minimal dissociation for the WT RBD-ACE2 interaction, global fitting across multiple concentrations produced an excellent fit (R²=0.9987), supporting the reliability of the derived kinetic and affinity values.

Minor:
Line 187: Typo “(Fi S4A-C)”

**Response**: Corrected.

Figure 1A, legend. I think the authors mean “Illustration” and not “Illusion”.

**Response**: Corrected.

**Part II – Major Issues: Key Experiments Required for Acceptance**

Reviewer #1:

1. The interaction between Fab-14 and two down RBDs is described as involving two interfaces: a primary interaction with residues 471-491 within the RBM and a secondary interaction with residues 331-335 of the neighboring down RBD. While the primary RBM interaction is well supported, the evidence for the secondary interface is comparatively weak. Specifically, the manuscript does not provide sufficiently resolved cryo-EM density at this interface to clearly support side-chain interactions or glycan involvement, nor are complementary binding assays (such as BLI assays using mutant RBD proteins) presented to directly validate this interaction. Although a slight reduction in neutralization activity is observed for the mutants incorporated with E484K, this alone does not convincingly establish a critical functional role for the proposed secondary site in the S-Fab-14 interaction. Moreover, the formation of such a secondary contact is, to some extent, understandable given the intrinsic conformational flexibility of RBDs (e.g., up and down configurations), which underlying the apparent structural tolerance among the three RBDs within the trimer. In this context, statements emphasizing a critical role of the secondary site, for example, “Site-directed mutagenesis and structural modeling validated the critical role of this secondary site in IgM-14-mediated neutralization”, appear to be overstated and should be reconsidered or more carefully qualified.

**Response**: We thank the reviewer for this point. This manuscript emphasized the functional importance of the secondary binding site to the IgM-14 neutralization. To address the reviewer’s point, we have revised the sentence to more cautiously reflect the supporting data: “Site‑directed mutagenesis and structural modeling revealed a critical role of this secondary site in IgM‑14-mediated neutralization.” This revised wording (lines 29-31) avoids overinterpretation.

2. The conclusion that IgM-14 can simultaneously engage Spike through multiple interfaces in diverse modes, thereby revealing a noncanonical avidity mechanism distinct from IgG-14, is an interesting hypothesis. However, this interaction model would benefit from more direct structural evidence at the IgM-Spike level. Cryo-EM or negative-stain EM data visualizing the intact IgM-14 in complex with Spike would substantially strengthen the claim of simultaneous engagement and better support the statements in the Abstract, such as “Unlike IgG-14, IgM-14 can simultaneously engage both interfaces in diverse modes” and “highlight the structural and functional uniqueness of IgM-14.”

**Response**: We thank the reviewer for this insightful comment. We fully agree with the reviewer that direct structural visualization of the intact IgM‑14-Spike complex would further strengthen the proposed model of simultaneous multivalent engagement. As noted, we made multiple attempts to obtain such structural evidence by cryo‑EM but were unable to resolve a high‑resolution IgM-Spike complex, largely due to sample heterogeneity/aggregation. Our negative‑stain EM analysis in concert with SEC data (newly added to the revision) indicated IgM‑induced spike aggregation and heterogeneity, which prevented us from capturing a well‑defined and interpretable architecture of IgM‑mediated cross-linking between multiple Spikes. Despite these technical limitations, several functional findings support the proposed mechanism of multivalent, noncanonical engagement. In particular, the neutralization data in the present study, together with our previously published ELISA‑based binding analyses demonstrating enhanced avidity and broad variant coverage of IgM‑14 compared to IgG‑14 (Ku, Z., Xie, X., Hinton, P.R. et al. Nature 2021), consistently indicate that IgM‑14 engages Spike through multiple interfaces in a manner distinct from the IgG isotype.

We have revised the manuscript text (lines 29-33) to more cautiously frame this conclusion: “Unlike IgG-14, structural modeling suggested that IgM-14 can simultaneously engage both interfaces in diverse modes, indicating a noncanonical avidity mechanism. Collectively, these findings highlight the structural and functional uniqueness of IgM-14 and offer valuable insights into the rational design of next-generation spike-targeted antibody therapeutics with enhanced breadth and potency.”

Reviewer #2:

1. Since there are two distinct binding sites on RBD, did the author observe the secondary binding event in BLI assay when the Fab associated to the immobilized RBD? Usually, the sensorgrams will display a heterogeneous binding mode for such a dual-binding event, which can be better fit with a bivalency model.

**Response**: We thank the reviewer for this excellent point. To test this, we conducted additional BLI analysis. Our results showed that Fab‑14 binding to RBD fit a 1:1 interaction model across low, medium, and high RBD surface densities (lines 317-321, Fig. S11). Increasing the RBD loading resulted in only minor changes in association rates and a slightly slower dissociation, with monophasic off‑rates in all cases. These patterns showed that the contribution of the secondary contact to the overall energetics of Fab-14 binding is negligibly small compared to that of the primary binding site.

2. If the secondary binding event can be observed in BLI, what is the kon/koff and KD?

**Response**: BLI did not reveal any measurable secondary binding phase, and as a result, kinetic parameters for the putative secondary interaction (*k*_on_/*k*_off_ and *K*_D_) could not be obtained.

3. Is there any synergism between the two binding sites?

**Response**: We were unable to directly detect synergistic engagement between the two binding sites using BLI. However, the neutralization data (Fig. 5G) clearly support functional synergy between the sites, most prominently in the IgM format. Substitutions at either site individually caused only modest reductions in neutralization potency (3‑fold for N331Q+L335A and 7‑fold for E484K), whereas simultaneous substitutions at both sites produced a dramatic, greater‑than‑additive loss of susceptibility (477‑fold). This pronounced combined effect is consistent with epistatic synergy between the two sites in driving IgM‑mediated neutralization.

4. Will the secondary binding site exclusively available in down conformation? Will there be clash or still compatible for the Fab binding to the site when RBD is up?

**Response**: The secondary epitope is accessible exclusively in the RBD‑down conformation. In the RBD‑up state, it becomes sterically occluded/incompatible with Fab engagement. Moreover, engagement of an up RBD expands the trimer, increasing inter‑RBD spacing so that bivalency by a single IgG is not feasible (please note: a single Fab cannot contact two up RBDs).

Reviewer #3: (No Response)

**Minor Issues**

Reviewer #1: Figure 1C: There appears to be no corresponding symbol “#” in the table, although it is mentioned in the legend. In addition, “Lota” should be corrected to “Iota.”

**Response**: Thank you for catching this error. Figure 1c has been corrected in the revision.

Line 199: The current naming of Mode V could be reconsidered. Among the five conformations described, the Spike structures can be broadly grouped into two categories: 1-up-RBD and 2-up-RBD states. Modes I-III all correspond to the 1-up-RBD conformation. Renaming Mode I as “1-up-RBD/1-Fab-1” and Mode II as “1-up-RBD/1-Fab-2,” for example, may help clarify the distinctions among these modes.

**Response**: Thank you for this helpful suggestion. We have named conformational modes in a way that follows the reviewer’s recommendation: Modes II and III both exhibit well‑resolved 1-up and 2-down RBDs. In contrast, Mode I contains two well‑resolved down RBDs and a third RBD that is relatively flexible. Because of this structural difference, we kept Mode I separate from Modes II and III.

Line 247: Cryo-EM density maps showing side-chain features of the interface residues from both Fab-14 and RBD should be provided to support the detailed description of the interface and proposed interaction forces.

**Response**: We have added three panels in Figure S5F-H in the revision to show the model-map overlays of the Fab-14-RBD interface at the primary, secondary binding sites.

Line 280: Similarly, cryo-EM density maps of side chains and glycans at the interface should be shown to better support the detailed structural interpretation of the interactions.

**Response**: As above, the model-map overlays have been provided in the revision.

Reviewer #2: 5. Minor point: label in Figure 3A seems wrong. The yellow ‘down RBD’ should be ‘up RBD’ instead.

**Response**: We confirmed the labeling of Figure 3A, which showed 2 down-RBDs bound by Fab. The third RBD was flexible and did not show reliable EM density. Both yellow and blue indicate down RBDs.

Reviewer #3: (No Response)
